# Influence of different thermopolymerization methods on composite resin microhardness

**DOI:** 10.4317/jced.56772

**Published:** 2020-04-01

**Authors:** Marlon-Ferreira Dias, Luís-Felipe Espíndola-Castro, Paulo-Cardoso Lins-Filho, Hilcia-Mezzalira Teixeira, Claudio-Heliomar-Vicente Silva, Renata-Pedrosa Guimarães

**Affiliations:** 1 Graduated in Dentistry from Federal University of Pernambuco; 2 Master student of the Dentistry postgraduate program of Federal University of Pernambuco; 3 Professor at Federal University of Pernambuco

## Abstract

**Background:**

Additional heat polymerization in composite resins allows greater effective­ness of microhardness, flexural strength, fracture tough­ness, wear resistance, and increased color stability.

**Material and Methods:**

150 composite resin specimens were made using a 4 mm diameter and 2 mm thick bipartite steel matrix. Five resins composed of different compositions were tested (Brilliant Everglow/Coltene, Filtek One BulkFill/3M, Filtek P60/3M, Filtek Z350XT/3M, Filtek Z250XT/3M), and for each of them three types of polymerization were tested: light curing only (n=50); photopolymerization + autoclave thermopolymerization (n=50) and photopolymerization + microwave thermopolymerization (n=50). Each specimen was submitted to three indentations by means of the Vickers microhardness test, applying a load of 300gf, associated with the time of 15s. Data were analyzed descriptively by means of statistics, standard deviation and coefficient of variation and inferentially by the F test (ANOVA) in the comparison between groups. The margin of error used in statistical test decisions was 5%.

**Results:**

The highest vicker microhardness averages were from the Control group (light curing only) on P60 (82.16) and Z250 XT (79.61) resins. The lowest averages were all verified on Brilliant Everglow resin in all polymerization methods studied: Photopolymerization (37.32), with microwave (43.80) and autoclave (45.12), followed by Bulk Fill 3M resin, ranged from 52.23 to 59.15.

**Conclusions:**

Both autoclave and microwave thermopolymerization methods showed similar behavior on the microhardness of the composites studied. Considering the resin type, there was a varied behavior compared to thermopolymerization, which increased the microhardness values for Brilliant Everglow resins (Coltene) and Filtek One Bulkfill (3M) and decreased for Filtek P60, Filtek Z350XT and Filtek Z250XT resins.

** Key words:**Dentistry, composite resins, polymerization.

## Introduction

Composite resins are materials of choice for posterior tooth restorations (1). This class of materials has aesthetic quality combined with satisfactory physical and mechanical properties (2). Therefore, it still has limitations such as polymerization contraction, difficulties in reestablishing proximal contact, inadequate resistance to abrasion and fracture in large areas of occlusal contacts and incomplete polymerization (3). However, clinical alternatives aimed at improving the properties of restorative material should be encouraged.

For direct restorations with composite resin, material’s polymerization is performed by visible light photopolymerization with an average wavelength of 470nm (4). This method has the advantage of being fast, safe and inexpensive (5). However, there are some limitations, such as the necessity to perform polymerization in small composite increments and the low and unequal conversion of monomers in different thicknesses of the restoration body (4).

Indirect restorations in composite resin are indicated in cases of major dental destruction (6). This technique allows for better proximal contact and restoration carving as it is not performed inside oral cavity (7). It minimizes stress induced by polymerization, or there is no plaster model and later compensated contraction during cementation (8). And it is possible to obtain an improvement in mechanical properties of the material through thermopolymerization (9).

Additional heat polymerization allows greater effectiveness of microhardness, flexural strength, fracture toughness, wear resistance, and increased color stability in restorative treatment (10). Additional curing treatment results in greater conversion of monomers into polymer chains (11).

Studies show that autoclave thermopolymerization is able to increase duration of restored parts (12,13). However, this step may not be possible to perform in the single clinical session due to the time of the autoclave cycle. Some clinical reports suggest that thermopolymerization can be performed by inserting restored parts immersed in water for 3 minutes in microwave ovens (5,14,15), which would facilitate this laboratory step. However, scientific evidence on this subject are scarce, further studies are needed to prove its effectiveness and applicability in clinical practice.

The objective of this study is to evaluate the influence of different polymerization methods on microhardness of composite resins. The null hypotheses tested are: (1) there is no difference between the different polymerization methods on microhardness of the different materials tested; (2) There is no influence of different types of composite resin on hardness.

## Material and Methods

The present work is an *in vitro* laboratory study, developed at the Multiple Materials Research Laboratory (LMPM) of the Pernambuco School of Dentistry (FOP / UPE) and at the Clinical and Biomaterials Research Center of the Federal University of Pernambuco (UFPE). Five resins of different compositions were tested (Table 1), and each resin group was divided into 3 subgroups depending on the type of polymerization employed: (1) light curing only, (2) light curing + autoclave, (3) light curing + micro, thus an amount of 15 subgroups (5 resins with 3 polymerization method for each). For each subgroup, 10 specimens were made, making a sample of n=150.

**Table 1:**
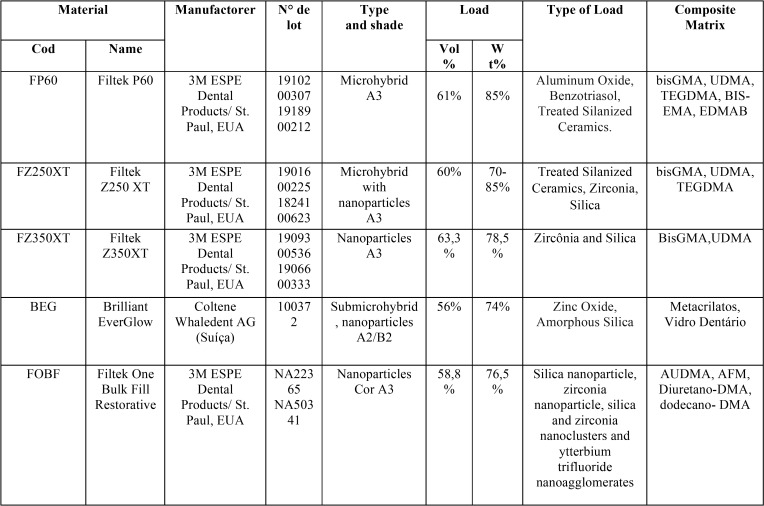
Description of the properties of the materials used in the research.

The composite resin specimens were made from a split steel matrix, 4 mm in diameter and 2 mm thick. The composites were inserted into the matrix on a polyester strip above a single-increment glass slide. Another polyester strip was placed over the resin inserted in the matrix and pressed by another glass slide to obtain a plane, smooth and polished surface. After the resin was inserted into the matrix and under pressure on the glass slide, light curing was performed for 20 seconds with a Radii-Cal high power LED (SDI) device. The light intensity of the device was measured by means of a radiometer (Hilux-LED) and maintained between 900 and 1,200 Mw / cm².

After light curing of all dimensions, additional curing was performed following the directions in Table 2.

**Table 2:**
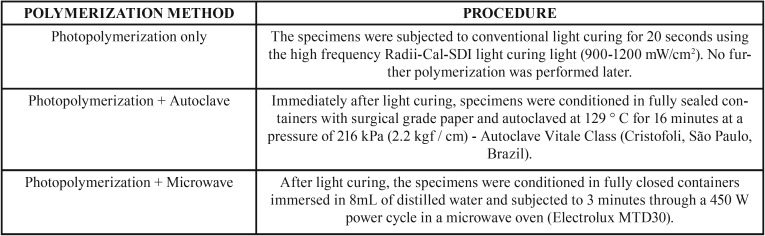
Procedure to each polymerization method.

After preparation, the specimens were stored in black closed containers, in order to prohibit any light passage to the made samples. These containers were filled with 8mL of distilled water inside and remained closed for 48 hours. After this time, the specimens were taken for analysis.

Each specimen was submitted to the Vickers microhardness test. In each specimen 3 measurements were made randomly by means of a square base diamond pyramid-shaped indentator, with an angle of 136o between the opposite faces, applying a load of 300gf, associated with the 15s time with the use of the Insize ISHV - D120 microdurometer (INSIZE - Loganville, Georgia, USA). The Vickers microhardness calculation for each specimen was obtained considering the average of the 3 indentations performed in each specimen.

Data were expressed as measurements: mean, standard deviation (mean ± SD) and coefficient of variation. For the comparison between the polymerization forms in each resin and the comparison between the resins in each polymerization method, the F test (ANOVA) was used, and if the difference was significant, Tukey’s multiple comparison tests were used in the case of. checking for variance equality or multiple size comparisons in case of rejection of equality of variances for the comparisons in question.

The choice of the F test (ANOVA) was due to the verification of the normal distribution in the data in each combination of resin and polymerization form. Normality was verified by the Shapiro-Wilk test and the equality of variances was by Levene’s F test. The margin of error used in deciding statistical tests was 5%. Data were entered into EXCEL spreadsheet and the program used to obtain statistical calculations was IMB SPSS version 23.

## Results

Table 3 presents the statistics: mean, standard deviation (mean ± standard deviation) and microhardness coefficient of variation according to resin type and polymerization method. In this Table we highlight the highest averages of the Control group (light curing only) on resins P60 (82.16) and Z250 XT (79.61). The lowest averages were all verified on Brilliant Everglow resin in all polymerization methods studied: Photopolymerization (37.32), with microwave (43.80) and autoclave (45.12), followed by Bulk Fill 3M resin. ranged from 52.23 to 59.15. For the fixed margin of error (5%), significant differences between the polymerization forms are shown. The multiple comparison tests found significant differences between the resins: in the light curing group, except between Z250 XT 3M and P60 3M resins; between all resin pairs when microwaves were used; except between Z350 XT 3M and Bulk Fill 3M resins and between Z250 XT 3M and P60, when the autoclave method was used. The expressed variability of the coefficient of variation has been shown to be greatly reduced since these values were at most 10.62% and therefore less than 33.3%.

**Table 3:**
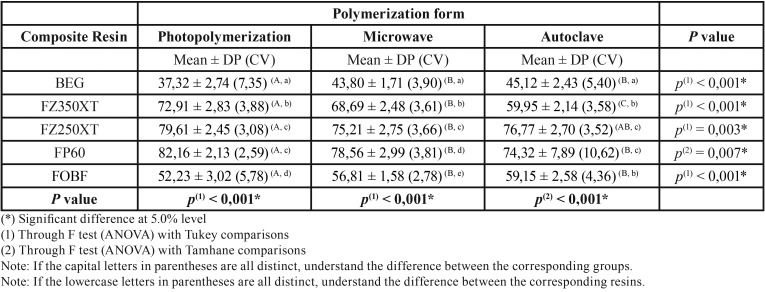
Statistics of microhardness by resin according to the polymerization method.

## Discussion

The first null hypothesis, that there is no difference between the different polymerization methods in the hardness of the different materials tested was rejected since there were statistically significant differences between the additional polymerization methods with the control group in all materials searched.

The second null hypothesis that there is no influence of different types of composite resin on hardness was also rejected, since statistically significant differences are observed between the researched materials treated with the same type of polymerization.

Hardness can be defined as the ability of a substance to resist an edentulator or penetrator. Knowledge of such mechanical property is important in dentistry, relating its results to the indication and clinical longevity of restorative materials (16). A high Vickers microhardness value of a restorative material is directly related to the durability of the restoration as it provides increased wear resistance, establishing a direct correlation between hardness and wear (17).

Among the existing tests for microhardness evaluation, the Vickers microhardness test is the most suitable for composite resins due to its higher stability and should preferably be used when checking the hardness of resin composites (17).

As observed in the present study, the composition of the restorative material directly influenced the hardness of the material. The mechanical properties of composite resins, considering the material composition, are related to the polymeric matrix (monomer composition), inorganic filler (type, size and distribution of filler) and binding agent (18). In the FP60 group, a higher hardness was observed when compared to the other groups (BEG; FZ350XT; FZ250XT; FOBF) in all types of polymerization. This may be related to the type (microhybrid) and charge particle size that is larger in the FP60 group. The lowest values were from the groups corresponding to BEG and FOBF resins, both of nanoparticulate technology, presenting smaller inorganic particle size when compared to microhybrid technology FP60. When low microhardness results are obtained, the risk of dissolution of the composite organic matrix, followed by exposure of the inorganic particles, increases the restoration roughness and the consequent accumulation of bacterial plaque, contributing to the restoration longevity decrease (19).

Higher temperatures present in the additional thermopolymerization increase free radicals and monomer mobility resulting in a higher resin matrix conversion and consequently higher microhardness values, which guarantees greater durability of the oral cavity restoration (20). As shown in Table 3, the BEG and FOBF groups had statistically significant hardness values when thermopolymerization was employed when compared to control groups where only light curing was performed.

In the present study, 2 types of additional thermopolymerization were analyzed together with the initial curing of 20s in 5 composites of different properties and clinical indications. It was found that autoclave and microwave thermopolymerization showed no significant change in microhardness values between the additional autoclave and microwave thermopolymerization types. These findings are similar to the results found by other studies (5,15), that compared the same additional thermopolymerization methods with the same time and power parameters as described in the present study.

Arossi and his contributors (5) evaluated the influence of additional polymerization on microhardness of direct and indirect restorative composites. In this work, the samples, after conventional 20 sec light curing, were subjected to the heat of the microwave oven with a power of 450W for 3 minutes, while in the autoclave a time of 7 minutes with a temperature equivalent to 134°C was used. No significant difference was found between the additional heat treatment groups, but higher microhardness values were achieved when compared to the conventional light curing method. Poskus (15) also analyzed the relationship of additional heat treatment to Vickers Microhardness. For the samples, only resins of hybrid technology were used, which were light cured for 40 sec and microwaved and autoclaved with the same parameters as the study by Arossi and his contributors (5). The study also concluded that there was no difference between the complementary light curing groups, however, when compared to the control group (40 sec light curing), the additional treatment obtained higher microhardness values.

In order to reduce clinical time in posterior teeth restorative procedures, bulk fill resins were introduced in the dental market. This composite category has gained popularity by simplifying and reducing clinical time, making the restorative method simpler (21). In the present study, the FOBF group, corresponding to Bulk Fill filtek one resin (3M), obtained lower vickers microhardness values in light curing (52.23) when compared to the groups: light curing + autoclave (59.15) and light curing + microwave (56.81). Similar results were also found in the BEG resin group, which recorded lower values in conventional polymerization (37.32), compared to the values presented in the groups corresponding to the additional heat treatment: autoclave (45.12) and microwave (43.80). Both composites present the nanohybrid technology, differing only in the resin matrix, where the FOBF group presents lower viscosity, consequently lower load, when compared to the BEG group resin, being, therefore, more viscous.

Additional polymerization through elevated temperatures promotes greater molecular mobility, increasing the microhardness value by converting the residual monomers into new polymer chains in the resin matrix (22). In addition, during the further heat polymerization process, about 1.3% of the organic portion of the matrix evaporates, providing a material with fewer constituents of poor properties and greater biocompatibility (5). A study (23) comparing different storage temperatures of bulkfill resins found an increase in vickers microhardness when samples were stored at a temperature equivalent to 35°C for a period of 24 hours.

A study on the influence of inorganic particle content on different material properties also showed that nanohybrid materials had lower microhardness values compared to microhybrid (24), as it corresponds to the values of the study, since the lower values of microhardness vickers were found in the nano technology feature groups (BEG, FOBF, FZ350XT).

This is due to the fact that materials with nano-charged particles have a higher surface area volume, which tends to absorb more water, and consequently, greater degradation of the organic matrix/charge particle interface (25). The decrease in vickers microhardness values can also be explained by the amount of load, the groups BEG, FOBF e FZ350XT have a lower amount of load when compared to the FP 60 and FZ250XT group, which have higher amount of inorganic load and higher microhardness values.

Ho and his contributors (26) analyzed by additional heat treatment, UDMA base materials showed higher water softening through temperature change when compared to base materials Bis-GMA. Result that may have influence in the present study, since the materials were stored in closed container containing water and subjected to elevated temperatures. Lower microhardness values were found in the BEG and FOBF groups, both nano technology and composed of a large amount of UDMA.

In materials where a high degree of conversion is found even in conventional photoactivation, there is little tendency for an increase in the degree of conversion after additional polymerization (16), as in the results found in the groups FP60, F250XT, F350XT.

This fact can also be explained by the use of the high frequency photoactivator apparatus (900-1200 mW/cm2), which was used in the preparation of the specimens.

Unlike the results of Loguercio (27) which justified an improvement in the mechanical properties of 3M ESPE Z350 resin over 3M ESPE P60 due to the addition of more UDMA and lessTEGDMA, in the present study P60 resin showed the best microhardness result, being composed of higher amount of TEGDMA, a result also found in a previous study (16).

The FP60 group has a microhybrid characteristic, this type of composite resin has in its composition two or more quartz and glass particle shapes between 0.2 and 3µm in size along with 5 to 15% of much smaller particles. The number of inorganic particles and their distribution bring to this type of composites some advantages, such as: high hardness, lower polymerization shrinkage and less water absorption (28).

For an increase in crosslink in the composite resin matrix to occur, this material must be subjected to an additional 150 ° heat treatment for at least 1 hour (29). In the present study, although specimens were subjected to additional heat polymerization, perhaps the time taken was insufficient to lead to statistically significant results. However, recent studies have shown increased microhardness vickers with parameters used in the present study (5,15).

A study that compared 5 polymerization methods, including autoclave and microwave thermopolymerization, a significant difference was found only in the group represented by the additional autoclave polymerization (14). Previous studies have not found statistically significant results regarding microhardness difference when subjected to additional autoclave polymerization tests in at least 1 of the analyzed groups (30,31). Although other authors in the literature argue that additional heat polymerization has beneficial consequences for the mechanical properties of composite resin (3,5,32,33).

Additional polymerization of direct composite resins is usually performed on specific dental office appliances such as the autoclave. In evaluating the efficiency of alternative complementary polymerization methods in direct resins, it can be observed in the present study that the additional microwave polymerization was efficient, increasing the microhardness of the previously cured composite, corroborating previous findings (5,15). There are still few studies that prove the use and effectiveness of microwaves in additional thermopolymerization, requiring further research in the area.

## Conclusions

1. Autoclave and microwave thermopolymerization methods showed similar behavior on the microhardness of the composites studied.

2. The studied resins showed a different behavior when compared to thermopolymerization, which increased the microhardness values for Brilliant Everglow (Coltene) and Filtek One Bulkfill (3M) resins and decreased for Filtek P60, Filtek Z350XT and Filtek Z250XT resins.
